# The Mechanism of Acupuncture in Treating Essential Hypertension: A Narrative Review

**DOI:** 10.1155/2019/8676490

**Published:** 2019-03-07

**Authors:** Juan Li, Mingsheng Sun, Jing Ye, Yuxi Li, Rongjiang Jin, Hui Zheng, Fanrong Liang

**Affiliations:** ^1^College of Health Preservation and Rehabilitation, Chengdu University of Traditional Chinese Medicine, Chengdu 610075, China; ^2^College of Acupuncture and Tuina, Chengdu University of Traditional Chinese Medicine, Chengdu 610075, China

## Abstract

Essential hypertension has a high incidence worldwide, and patients with essential hypertension endure a lifetime of medication, leading to a heavy economic burden on the patient's family and causing serious impacts on the patient's quality of life. Much evidence has demonstrated that acupuncture as an adjunctive therapy can lower blood pressure in patients with hypertension, but the mechanism of its action is unclear. This article reviews the research from 2000 to 2018 regarding the mechanism of acupuncture for hypertension, and we summarize the current knowledge about using acupuncture for hypertension. We found that the mechanism whereby acupuncture lowers blood pressure is related to the regulation of renin-angiotensin-aldosterone system, vascular endothelium, oxidative stress, neuroendocrine system, and so on. Besides, there may be cross-talk between multiple systems and multiple targets. We also investigate the influence factors of acupuncture for hypertension. These results may provide evidence and research ideas for the treatment of hypertension via acupuncture.

## 1. Introduction

According to the* 2017 Guideline for High Blood Pressure in Adults *[1], hypertension is one of the most frequent cardiovascular diseases, and its main manifestation is elevated blood pressure (BP) (systolic BP≥130 mmHg or diastolicBP≥80 mmHg) in adults [1]. Hypertension affects approximately one billion individuals worldwide. In the United States, about 46% of adults suffer from hypertension, and its morbidity and mortality are increasing year by year [1, 2]. In 2008, the estimated direct and indirect costs of hypertension were $69.9 billion in the United States, leading to a substantial economic burden on healthcare systems [3]. It is reported that approximately 62% of strokes and 49% of myocardial infarctions are caused by high BP[4]. Additionally, other common complications (for example, hypertensive encephalopathy [5], hypertensive coronary heart disease [6], atherosclerosis [7], and renal failure [8]) often come with hypertension. The economic loss caused by hypertension and its complications is a heavy burden for both patients and their families. Hypertension has seriously hampered socioeconomic development and social stability and poses a huge threat to the social health structure. At present, the treatment of hypertension is mainly via drugs and lifestyle modifications [9]. Despite their proven benefits, using these methods to achieve an ideal BP level in patients with hypertension is not satisfactory, because it requires a combination of multiple drugs, which may confer increased risk of side effects [10–12]. At the same time, numerous adverse reactions, including headaches, dizziness, orthostatic hypotension, and decreased sexual function, limit the clinical practice of using antihypertensive drugs [10, 11]. Nonpharmacological treatments are recommended in the management of hypertension [1]. Therefore, an increasing number of researchers and patients are beginning to seek a variety of these nonpharmacological treatments, including acupuncture. Acupuncture is able to lower BP, resulting in medication dosage reduction, medication types reduction, and fewer side effects, which is regarded as an adjunctive therapy for hypertension.

Acupuncture is one of the most widely practiced forms of nonpharmacological treatments. According to up-to-date clinical studies [13–15], acupuncture treatment exerts a good effect in lowering BP, and so its usage has attracted increased attention. The results of our previous clinical study have found that, after acupuncture treatment, there is a decrease in 24-hour systolic and diastolic BP (from 145.10 ± 9.28 mmHg to 140.70 ± 9.59 mmHg [*P*< 0.0001], and 88.35 ± 7.92 mmHg to 85.86 ± 7.95 mmHg [*P* = 0.0024], respectively) and an improvement in circadian BP rhythm in patients with mild hypertension [16]. However, the mechanism of acupuncture in treating hypertension remains unclear. Therefore, the aim of this article is to review the research over recent years into the mechanism of acupuncture in treating hypertension and to summarize the current knowledge regarding the use of acupuncture for hypertension.

## 2. Acupuncture Treat Primary Hypertension

### 2.1. Influence of Acupuncture on the Renin-Angiotensin-Aldosterone System

The renin-angiotensin-aldosterone system (RAAS) can regulate BP homeostasis, vascular injury, and repair responses and is associated with inflammation, fibrosis, and target-organ damage [17]. Acupuncture lowers BP by decreasing the activity of rennin [18, 19], demonstrating that the effect of lowering BP is partly due to a decrease in renin secretion. Angiotensin II (Ang II), which is catalyzed by rennin and angiotensin in converting enzyme (ACE), has the effect of contracting blood vessels and thus increasing BP. A large number of animal experiments have shown that acupuncture can lower BP by decreasing the levels of ACE and Ang II receptors (AT1R, AT2R) and decreasing the plasma content of Ang II [13, 20–22]. In addition, acupuncture can significantly decrease the mRNA expression of angiotensinogen (AGT) and AT1R in the aorta [23]. Ang II can promote the secretion of aldosterone, which can regulate blood volume. The plasma concentration of aldosterone was decreased after acupuncture treatment [15]. Luo [24] found that acupuncture can reduce the activity of renin and the concentrations of angiotensin and aldosterone in patients with essential hypertension. In summary, the antihypertensive mechanism of acupuncture may be related to the regulation of the hormones, their corresponding enzymes, and receptors of RAAS.

### 2.2. Effect of Acupuncture on Oxidative Stress

Vaziriet al. [25] reported that oxidative stress caused by glutathione (GSH) depletion led to a marked elevation in BP. In turn, the antioxidative treatment can reduce the elevated BP but has no effect on the BP of healthy rats; this proves that oxidative stress is considered a mechanism for hypertension [26]. Antioxidative treatment is essential for hypertension, as it can reduce the formation of ROS, regulate the oxidation/antioxidant enzyme system [27], and improve the body's antioxidant defense capabilities [28]. Overproduction of reactive oxygen species (ROS) has been proven to be essential in the pathogenesis of hypertension [29]. ROS, derived from nicotinamide adenine dinucleotide phosphate (NADPH) oxidases (Nox), is important in regulating endothelial function and vascular tension [29]. Recent studies showed that acupuncture-reduced BP may be due to the inhibition of Nox, enhancing antioxidant capacity and reducing the level of ROS [20, 30]. Liu et al. [27] explored the mechanism of acupuncture in reducing BP from the perspective of oxidative/antioxidant enzymes, and their results demonstrated that acupuncture could reduce Nox, increase catalase (CAT), and inhibit oxidative stress after needling Renying (ST9). Superoxide dismutase (SOD) is one of the most important antioxidants, which can effectively remove ROS and maintain its dynamic equilibrium by catalyzing the redox reaction of superoxide anion radicals. Increased SOD can promote the enhanced antioxidant function, relaxing vascular smooth muscle and lowering BP. Lai et al. [31] found that the mechanism whereby acupuncture lowers BP may increase the antioxidant enzymes (SOD, glutamate dehydrogenase 1, aldehyde dehydrogenase 2, glutathione S-transferase M5, Rho GDP dissociation inhibitor 1, and DJ-1 protein) in the medulla and improve oxidative stress, by needling Taichong (LR3) of spontaneously hypertensive rats (SHR). In conclusion, acupuncture reduces the formation of ROS by regulating the oxidation/antioxidant enzyme system for the treatment of essential hypertension.

### 2.3. Influence of Acupuncture on Vascular Endothelial Function

Animal research has confirmed that the endothelium-dependent diastolic blood vessels in hypertensive animal models were damaged, which suggested that endothelial dysfunction may be one of the etiologies of hypertension [32]. Studies have shown that the dynamic balance of endothelium-dependent relaxing factor (EDRF) and endothelial cell-dependent contractility factor (EDCF) produced by vascular endothelial cells is essential for maintaining normal vascular tension [33, 34]. If the dynamic equilibrium of EDRF and EDCF is broken which would lead to disorders of vascular tension and elevation of BP, it has been reported that acupuncture is able to improve endothelial dysfunction and the diastolic function of blood vessels [35]. Animal studies have shown that acupuncture can lower BP by reducing endothelin A receptor (ETAR) and ET-1 production [21, 22, 36]. Kim et al. [37] reported that the expression of endothelial nitric oxide synthase (eNOS) and neuronal nitric oxide synthase (nNOS) and production of NO were enhanced by electro-acupuncture (EA) on Zusanli (ST36) and confirmed that the activation of nitric oxide synthase (NOS) may be one of the antihypertensive mechanisms of acupuncture. Research has shown that acupuncture can regulate the NOS function of vascular endothelial cells, promote the production of NO, and decrease vascular resistance [38] and also that EA inhibits the activity of Nox, enhances antioxidant capacity, increases the production of eNOS and phosphorylated eNOS, and increases NO bioavailability in SHR [20]. Meanwhile, researchers [39] have also found that acupuncture instead of noninvasive sham acupuncture can increase the content of NO in healthy volunteers. To sum up, acupuncture treatment can lower BP by restoring the equilibrium of EDRF and EDCF in vascular endothelial cells.

### 2.4. Effect of Acupuncture on Inflammatory Factors

Clinical studies have shown that inflammation exists in hypertensive patients [40–43]. Inflammation is involved in the pathogenesis of hypertension and interacts with hypertension [44]. C-reactive protein (CRP), a marker of inflammation, is considered to be significantly associated with the occurrence and progression of hypertension [45–49]. It has been reported that CRP reduces NO produced by endothelial cells [50], upregulates the expression of AT1R [42], and downregulates the expression of AT2R [51], influencing the RAAS and contributing to hypertension. Besides, CRP can induce the expression of adhesion molecules in human endothelial cells [52]. Upregulation of adhesion molecules is a part of the inflammatory response. Cottone et al. [53] found that subjects with essential hypertension had higher levels of 8-iso-prostaglandin-F2*α* (8-iso-PGF2*α*), CRP, intercellular adhesion molecule–1 (ICAM-1), vascular adhesion molecule–1 (VCAM-1), and tumor necrosis factor–*α* (TNF-*α*) than healthy subjects and confirmed the relationship of CRP with systolic BP. Our previous review concluded that the antihypertensive effect of acupuncture was mediated by the reduction of inflammatory factors (TNF-*α*, interleukin-6, and CRP [54]) in blood [55]. All the evidence suggests that acupuncture can reduce inflammatory factors, which may affect the RAAS system and endothelial function, resulting in reduction of BP.

### 2.5. Influence of Acupuncture on Neuroendocrine

Research has shown that the antihypertensive effect of acupuncture may be related to the neuroendocrine system. The hypothalamus is one of the antihypertensive targets, and it has been reported that the paraventricular nucleus (PVN) and arcuate nucleus (ARC) in the hypothalamus can regulate cardiovascular function [56–58]. Research found that EA reduces activity in the rostral ventrolateral medulla (rVLM) through an opioid mechanism in the PVN that is rich in endorphinergic fibers and *μ*-opioid receptors, contributing to the prolonged inhibition of cardiovascular reflex responses by EA [59–61]. The ARC can secrete glutamate, acetylcholine, and other neurotransmitters and the ARC is rich in *β*-endorphinergic neurons [57, 62]. The *β*-endorphinergic arcuate neurons might be involved in the inhibitory action of EA on cardiovascular excitatory responses [63]. Furthermore, activation of the ARC also activates PVN opioid receptors [57, 62] and reduces activity in the rVLM. The neurons in the ARC are rich in endorphins and project directly to the rVLM, suggesting they might activate *μ*-opioid receptors and consequently inhibit rVLM neurons [64]. The ventrolateral periaqueductal gray (vlPAG) and the rVLM in medulla oblongata also play an important role in the regulation of cardiovascular function.

The vlPAG and the rVLM in medulla oblongata also play an important role in the regulation of cardiovascular function. Among them, rVLM plays a key role in the regulation of BP [65]. Inhibition of neuronal function in rVLM results in a drastic reduction in BP [66]. Opioids and gamma-aminobutyric acid (GABA) in rVLM are the specific neurotransmitters for cardiovascular regulation [64, 67, 68]. The research found that acupuncture can inhibit rVLM premotor sympathetic cardiovascular neurons [69] and can attenuate visceral sympathoexcitatory reflexes through opioid-mediated inhibition of aspartic acid (Asp) and glutamic acid (Glu) action in the rVLM [70]. Administration of naloxone (nonspecific opioid receptor antagonist) or xylazine (gamma-aminobutyric acid or GABA type A receptor blocker) in rVLM abolished EA modulation [71]. Acupuncture can affect the sympathetic outflow and cardiovascular function through the enkephalin mechanism [72]. Endorphinergic ARC projections combined with enkephalinergic vlPAG and reciprocal projections comprise the long-loop pathway that eventually inhibits rVLM neurons, and which is critical for the sympathoinhibition occurring during EA [63]. Glutamate, opioids, GABA, and endocannabinoids in the medulla oblongata are shown to be involved in the antihypertensive response of EA. Central effects may also affect the endocrine system and lead to a decrease of aldosterone, Ang II, and norepinephrine in plasma. Therefore, the mechanism of acupuncture's beneficial effects is related to the sympathetic outflow and regulation of the endocrine system [73]. Also, a study [74] showed that the complex interaction between the autonomic nerve center of brain stem and the antiopiate stimuli and other chemical mediators determines the degree of influence of acupuncture on BP. Therefore, acupuncture works through the activation of the PVN, ARC, and vIPAG, together inhibiting rVLM and resulting in an antihypertensive effect.

### 2.6. Influence of Acupuncture on Brain Functional Imaging

Brain functional imaging evidence has shown that brain function may be changed in hypertensive patients, and some brain regions are associated with changes in BP. Gianaros et al. [75, 76], using functional magnetic resonance imaging (fMRI), found that higher mean arterial BP correlated with greater blood oxygen level–dependent activation in two regions of the cingulate cortex (perigenual and midanterior) and other networked brain regions, including the insula, thalamus, and periaqueductal gray. The regulation of acupuncture is mediated by the midbrain and brainstem network, including the hypothalamus, medulla oblongata, lateral ventral region of midbrain periaqueductal gray, and dorsal medial prefrontal cortex [77]. The hypothalamus was found to be the most important part of the brain in controlling the autonomic nervous system [78]. Its corresponding brain network showed enhanced connectivity with the medulla, brainstem, cerebellum, margin system, thalamus, and frontal lobe function after acupuncture. Needling LR3 can lower BP and improve symptoms by activating the anterior cingulate gyrus to regulate the parasympathetic nerve and also improve the cognitive impairment caused by long-term hypertension by strengthening the connection of the anterior cingulate gyrus with the function of other brain regions [79]. Wang et al. [80], using resting-state fMRI, found that the limbic system (insula, parahippocampal gyrus, and cingulate cortex), cerebellum, bilateral thalamus, and frontal lobes may be targets in the brain, by needling LR3 and Taixi (KI3), and maintained their original treatment in hypertensive patients. Wang et al. using ^18^F-2-fluoro-deoxy-D-glucose positron emission tomography (^18^F-FDG-PET) demonstrated the influence of acupuncture at LR3 altered cerebral glucose metabolism in the hypothalamus, thalamus, medulla oblongata, and cerebellum [81]. Therefore, acupuncture can regulate the cortical level, the hypothalamus, and the brain stem, along with other complex brain networks, so as to reduce the autonomic nervous response [77] and regulating the cardiovascular system [15, 82, 83], thus resulting in an antihypertensive effect.

### 2.7. Influence of Acupuncture on Metabolism in Essential Hypertension

A cross-sectional survey showed that the prevalence of metabolic disorders is almost 60% in the hypertensive population [84]. Hypertension is often associated with various metabolic abnormalities, such as elevated triglycerides, reduced high-density lipoprotein, glucose intolerance, insulin resistance, and so on [85]. Hypertension due to metabolic disturbances can be defined as metabolic hypertension [86]. Wang et al. [87] found that acupuncture can lower BP in SHR and, at the same time, improve metabolic disturbances, such as increased urinary metabolites *α*-ketoglutarate, N-acetylglutamic acid, and betaine. Our recent research [16] found that oleic acid (OA) and myoinositol (MI) were the most important differential metabolites between the hypertensive plasma and the healthy plasma. The correlation analysis showed that SBP reduction is positively correlated with OA change (R=0.44) and negatively correlated with MI change (R=−0.20). Acupuncture simultaneously lowered 24-hour BP and reversed OA and MI abnormalities. Therefore, OA and MI can be used as biomarkers for preliminary assessment of the effect of acupuncture treatment for hypertension. OA is one of the most important free fatty acids (FFAs) that is, considered to be associated with higher cardiovascular risk induced by FFA-related oxidative stress in endothelial cells. OA can increase the production of mitochondrial ROS and decrease the activity of endothelial nitric oxide synthesis [88]. The possible mechanism of MI attendant in regulating BP may be through inositol 1,4,5-triphosphate receptor (IP3R). IP3R upregulation in hypertension is associated with sensitization of Ca^2+^ release and vascular smooth muscle contractility [89]. Metabolic research around acupuncture treatment of hypertension has not been widely carried out. The evidence mentioned above indicates that the improvement seen in the metabolism with the use of acupuncture may be one of the mechanisms of antihypertension.

### 2.8. Influence of Acupuncture on Genes

A large amount of researches into the mechanisms of genes in hypertension has recently been conducted. Guo et al. [90], in a study based on gene chip technology, found the overexpression of genes in the heart of the stress-induced hypertensive rat, including heat shock 70 kDa protein (HSPA1A), heat shock protein beta–1 (HSPB1), oxidized low-density lipoprotein receptor 1 (OLR1), phospholipase A2 (PLA2G4A), and prostaglandin-endoperoxide synthase 2 (PTGS2), which are related to the contraction of vascular smooth muscle and may be specific expression genes for hypertension. The research also found that EA can lower BP by downregulating the expression of the genes mentioned above. Similarly, Xie et al. [91] reported the overexpression of genes (HSPB1, protein phosphatase 1 regulatory subunit 14A, and tyrosine hydroxylase (TH)) in the hypothalamus of the stress-induced hypertensive rat. HSPB1 is related to the contraction of vascular smooth muscle; protein phosphatase 1 regulatory subunit 14A (Ppp1r14a) and P2X purinoceptor 4 (P2RX4) are related to the regulation of Ca^2+^ concentration; TH is related to sympathetic nerve excitability. The expression of these genes was significantly higher in hypertensive rats when compared with normotensive rats, while EA downregulated the expression of the genes mentioned above. Another study found that EA is able to downregulate the mRNA expression of AGT, AT1R, ET1, and ETAR in SHR rats, as detected by real-time quantitative polymerase chain reaction (RT-PCR) [23]. There are few studies focusing on the genomic mechanism of antihypertensive acupuncture. However, with the development of gene technology, acupuncture is expected to reveal its mechanism on the basis of the genes involved.

### 2.9. Influence of Acupuncture on Target Organ Damage

Hypertension can cause damage to the target organ (such as the heart, kidneys, and brain). ACCF (American College of Cardiology Foundation)/AHA (American Heart Association) guidelines [92] recommend assessing the degree of damage to the target organ by measuring renal function and proteinuria and performing an electrocardiogram. Research has found that the incidence of cardiovascular events is still high even when the BP in hypertensive patients is under control. This may be due to target organ damage, and therefore the treatment of hypertension involves not only focusing on reducing BP but also reversing the damage to the target organ [93]. Acupuncture can significantly improve cardiac hypertrophy and the thickness of the aortic wall in order to prevent new vascular remodeling in SHR [22, 94, 95]. The latest clinical study unveils the cardioprotective effects of acupuncture through improving wave reflection (augmentation index, AIx) and arterial stiffness in hypertensive patients [96]. This indicates that acupuncture can protect the heart by improving cardiac hypertrophy and dysfunction [22, 94, 97]. Studies have reported that acupuncture can improve cerebral blood flow and increase microvascular openings in the hippocampus CA1 area by increasing the proportion of Bcl-2/Bax and inhibiting apoptosis, in order to lower BP and prevent target organ damage [21, 98]. Ang II activates the production of ROS derived by Nox, leading to the damage of renal tubules and blood vessels [99–103]. Acupuncture can protect blood vessels and renal tubules by inhibiting Ang II and reducing Nox. Acupuncture can also reduce the sympathetic excitability, improve renal function, and decrease 24-hour urinary protein in order to protect the kidney [104, 105]. Therefore, acupuncture treatment can protect the target organ by regulating the RAAS, oxidative stress, and the inflammatory reaction.

## 3. Discussion

Acupuncture as an adjunctive therapy has been proven to be an effective nonpharmacological therapy for hypertension [15, 16]. Additionally, the antihypertensive effect of EA persists for at least a month after the end of EA treatment [15]. Acupuncture as a nonpharmacological therapy has fewer side effects, in contrast to drug treatments. Acupuncture can not only lower BP but can also protect the target organ. A clinical study found that acupuncture combined with their original treatment can reduce the number of patient complaints and also improve patient compliance [106]. In summary, acupuncture as a treatment for hypertension has many advantages, in terms of efficacy, sustained effects, fewer side effects, target organ protection, improvement in patient compliance, etc. We have reviewed the research from 2000 to 2017 studying the mechanism of acupuncture in treating hypertension and have found that RAAS, vascular endothelium, oxidative stress response, neuroendocrine system, genes, metabolism, and other factors are all involved in the antihypertensive mechanism of acupuncture. In addition, there is cross-talk between multiple systems and multiple targets. For example, acupuncture may increase NO to regulate endothelial function, also downregulate the expression of AT1R [42], and upregulate the expression of AT2R [51] to affect RAAS by regulating CRP [54]; or acupuncture may inhibit the oxidative stress of ROS by inhibiting Ang II [20, 99, 102], resulting in antihypertensive effect. In addition to the antihypertensive effect, acupuncture also can protect target organs by improving the endothelial function and inflammatory response.

**Figure 1:**
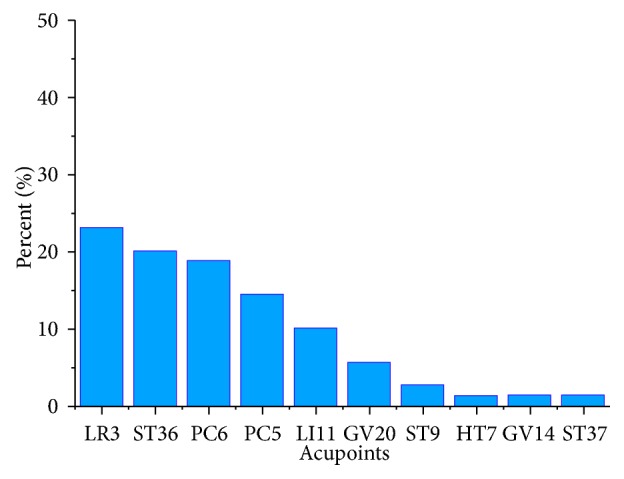
Acupoint selection in essential hypertension. Taichong(LR3), Zusanli(ST36), Neiguan(PC6), Jianshi(PC5), Quchi(LI11), Baihui(GV20), Renying(ST9), Shengmen(HT7), Dazhui(GV14), and Shangjuxu(ST37).

The animal study and clinical trial has demonstrated that sham acupuncture treatment (acupuncture on the nonacupoints) could not lower BP [107, 108].When irrelevant acupoints (e.g., LI6, LI7, GB37, GB38, and GB39) were selected, the antihypertensive effect is not achieved too [15]. Thus, acupoint selection is one of the main factors that affect the antihypertensive effect of acupuncture. We review all the mechanism researches of acupuncture for hypertension from 2000 to 2018; it is found that LR3, ST36, and Neiguan (PC6) are the most common used acupoints in the treatment of hypertension (Figure 1). Except for single acupoint selection (41.86%), multiple acupoints were often chosen (58.14%), among which, LR3 and Quchi (LI11), PC6 and Jianshi (PC5), and ST36 and LI11 are often used in conjunction. However, whether different acupoints application or different multiple acupoints are used in conjunction may contribute to different antihypertensive mechanism.

We also found that EA is the commonly used intervention method among antihypertensive researches (69.77%). The reason may be that the stimulus quantity and intensity of EA are stable and controllable and can eliminate the interference of manipulation practices of manual acupuncture. Whether there is any different of antihypertensive mechanism between EA and manual acupuncture is unknown, which needs further study. The intensity and frequency of EA play an important role in blood pressure regulation. It is generally agreed that higher intensity EA has excitability adjustment, which leads to the elevation of blood pressure; in contrast, lower intensity EA has inhibitory effect, which may result in blood pressure reduction [109, 110]. However, the influence of intensity of EA on blood pressure is unknown yet. The frequency of EA in hypertension treatment is mostly 2Hz (73.33%). It has been confirmed that low frequency EA (2Hz) can induce a decrease in sympathetic tone, cause dilatation of the systemic arteriole, and result in depressor response on BP [111]. Thus the selection of EA parameters is an important factor for EA in the hypertension treatment.

SHR (48.84%), visceral reflex induced hypertension animal models (23.26%), and stress-induced hypertension animal models (16.28%) are the commonly used animal model in the mechanism researches of acupuncture for hypertension. SHR is a natural animal model of essential hypertension, of which the systolic pressures can reach up to 180-200 mmHg when SHR in the adult age phase. Visceral reflex induced hypertension animal models are developed by the stimulation of gallbladder or stomach or splanchnic nerve [112]. For the stimulation of gallbladder or stomach, a latex balloon was inserted into the gallbladder or stomach and the balloon was inflated inside the gallbladder or stomach to induce elevation in BP. As for splanchnic nerve stimulation, the splanchnic nerve was isolated and stimulated with a Grass stimulator (0.2–0.4 mA, and 0.5 ms pulses at 2 Hz) to induce a reflex increase in BP. All the visceral reflexes mentioned above can lead to stimulation of the sympathetic nervous system through activation of cardiovascular premotor sympathetic neurons in the rVLM. Stress-induced hypertension animal model is developed by repeated exposure to acute stress (such as electric foot shocks, noise, cold etc.), which is able to enhance sympathetic nerve system inducing hypertension [113]. However, the antihypertensive mechanisms of acupuncture may vary in different models.

In summary, the mechanism researches at present addressed the question of how acupuncture can treat hypertension. However, there are still some more questions need further study. For example, what is the optimal acupoint program for hypertension? And what is the corresponding mechanism? What is the optimal acupuncture stimulus for hypertension? And what is the corresponding mechanism? New research technology should be used to answer the questions in the future.

## 4. Conclusion

Acupuncture as an adjunctive therapy has antihypertensive effect. The mechanism of acupuncture for hypertension is related to RAAS, vascular endothelium, oxidative stress, the neuroendocrine system, and other factors; also, there is cross-talk between multiple systems and multiple targets. In addition, the functional images, genes, metabolism, etc. change with antihypertensive effect. Acupuncture can protect target organs in addition to reducing BP. Overall, based on the mechanism research acupuncture may be an effective intervention for management of hypertension.
